# Effect of Midazolam Premedication on Salivary Cortisol Levels in Pediatric Patients with Negative Frankl Behavior: A Pilot Study

**DOI:** 10.3390/children12081097

**Published:** 2025-08-20

**Authors:** Juan Ignacio Aura-Tormos, Laura Marqués-Martínez, Esther Garcia-Miralles, Isabel Torres-Cuevas, Bianca Quartararo, Clara Guinot-Barona

**Affiliations:** 1 Faculty of Medicine and Health Sciences, Catholic University of Valencia, San Vicente Martir, 46001 Valencia, Spain; j.ignacio.aura@ucv.es (J.I.A.-T.); esther.garcia@ucv.es (E.G.-M.); bianca.quartararo@mail.ucv.es (B.Q.); clara.guinot@ucv.es (C.G.-B.); 2 Dentistry Department, Faculty of Medicine and Dentistry, University of Valencia, 46010 Valencia, Spain; 3 Physiology Department, University of Valencia, 46010 Valencia, Spain; maitocue@uv.es

**Keywords:** premedication, salivary cortisol, child behavior, sedation

## Abstract

**Highlights:**

**What are the main findings?**

- Midazolam premedication in pediatric patients with “definitely negative” Frankl behavior was associated with significantly lower post-treatment salivary cortisol levels compared to non-sedated patients.
- Invasive dental procedures produced a marked increase in cortisol levels, while non-invasive treatments did not significantly alter cortisol concentrations.

**What is the implication of the main finding?**

- Midazolam may help reduce physiological stress in uncooperative pediatric dental patients, particularly during invasive procedures.
- Salivary cortisol measurement is a feasible and non-invasive biomarker for as-sessing stress and evaluating the impact of behavioral and pharmacological inter-ventions in pediatric dentistry.

**Abstract:**

Background/Objectives: This pilot study aimed to evaluate stress levels in pediatric patients classified as definitely negative according to the Frankl scale by measuring salivary cortisol concentrations. Additionally, the study assessed the impact of Midazolam premedication on stress reduction during dental procedures. Methods. Children and adolescents attending the Pediatric Dentistry Master’s program at the Catholic University of Valencia participated in the study. Salivary cortisol levels were measured before and after dental treatments, differentiating between invasive and non-invasive procedures. Patients were divided into two groups: those receiving Midazolam premedication and those who did not. Results. Findings showed a significant increase in cortisol levels following invasive dental treatments (0.991), whereas non-invasive treatments (0.992) did not lead to notable changes (*p* < 0.001). Patients premedicated with Midazolam exhibited significantly lower post-treatment cortisol levels compared to those who did not receive the medication (*p* < 0.05). Conclusions. These preliminary findings suggest that Midazolam-based management in children with definitely negative behavior may be associated with reduced physiological stress responses. As a pilot study with a limited sample and inherent group-allocation bias, the results should be interpreted with caution. The methodology proved feasible and supports the use of salivary cortisol in future, larger-scale studies designed to disentangle behavioral and pharmacological effects.

## 1. Introduction

Pediatric dental care requires attention to clinical, psychological, and physiological aspects influencing patient experience. Anxiety in children is common and crucial to manage for effective treatments [1,2,3]. The Frankl scale is widely used to assess children’s behavior in dental settings, with “definitely negative” ratings indicating high dental fear and posing challenges for behavior management. Despite the recognized importance of dental anxiety, its biological underpinnings remain underexplored, and identifying reliable biomarkers—such as salivary cortisol—could enhance targeted interventions [3,4].

This research addresses the need to integrate subjective and biological aspects of anxiety in pediatric patients during dental visits, focusing on the relationship between Frankl scale ratings and salivary cortisol levels.

Dental behavior management problems (DBMP) affect about 23% of children, leading to dental fear and anxiety (DFA) and impacting their treatment tolerance. DFA refers to excessive, irrational emotional states during dental procedures, often linked with local anesthesia, age, negative experiences, and pain. Proper management of DFA is crucial for high-quality dental care [5]. Children’s anxiety and cooperation levels vary, peaking between ages 3 and 7, then decreasing with age [6]. Positive experiences can reduce dental fear and improve oral health-related quality of life [7]. Dentists must ensure effective treatment and instill positive attitudes in children [6].

Three methods exist to evaluate dental anxiety: behavioral assessment, psychological assessment through questionnaires, and physiological response analysis, including salivary cortisol levels [8]. The Frankl scale categorizes children’s behavior from definitely negative (− −) to definitely positive (++), aiding daily pediatric dental practice [9].

Advanced behavior management approaches include sedation, protective stabilization, and general anesthesia [5]. Sedation levels vary from minimal (anxiolysis) to dissociative, each with specific requirements. Sedation in pediatric dentistry involves administering medications to facilitate procedures while maintaining airway reflexes and reducing anxiety and pain [10].

Common sedation methods for children with DBMP and DFA include oral Midazolam and nitrous oxide. Midazolam, a short-acting benzodiazepine, offers rapid onset and multiple administration routes, with oral and intranasal being most popular for their effectiveness and acceptance [11,12,13,14,15].

Evaluating sedation efficacy involves physical measures like blood pressure and biochemical markers like salivary cortisol, a non-invasive, simple method reflecting anxiety levels. Stress activates the hypothalamic–pituitary–adrenal (HPA) axis, increasing blood cortisol, peaking 20–30 min after stress [5,13,14,15,16,17,18]. Cortisol follows a circadian rhythm, peaking in the morning and declining throughout the day, influenced by genetic and environmental factors [16,17,18,19,20,21,22].

We hypothesized that pediatric patients classified as definitely negative on the Frankl scale and receiving oral Midazolam would show significantly lower post-treatment salivary cortisol concentrations compared to patients who did not receive the medication.

This pilot pragmatic study evaluates salivary cortisol variation in a real-world clinical scenario in which premedication with Midazolam is indicated for children with definitely negative Frankl behavior and compared with children with positive Frankl behavior managed without sedation. Therefore, the results reflect differences between management strategies rather than the isolated pharmacological effect of Midazolam.

The decision to include both Frankl-positive and Frankl-negative children was based on the pragmatic nature of pediatric dental care. Children with positive behavior usually do not require pharmacological support and therefore represent the real-world comparison group for those with definitely negative behavior who are clinically indicated for sedation. This design provides clinically meaningful contrasts between common management strategies, even though it does not allow the isolation of the pure pharmacological effect of Midazolam.

## 2. Materials and Methods

### 2.1. Study Population

The sample size of 50 participants, including males and females, was determined through an a priori power analysis using standard statistical methods. This analysis, based on a two-tailed independent samples *t*-test, was designed to ensure a statistical power of 80% and a significance level of 5% (α = 0.05) to detect clinically meaningful differences in salivary cortisol levels between the premedicated (test) and non-premedicated (control) groups. Salivary cortisol was selected as a validated and non-invasive biomarker for assessing physiological stress in pediatric populations. This methodological approach strengthened the reliability and reproducibility of the results (Table 1).

Patients included in the study were classified according to the Frankl behavior scale. Twenty-five patients with definitely negative Frankl behavior were assigned to the premedication group and received oral Midazolam. The remaining 25 patients, classified as Frankl positive, constituted the control group and did not receive sedation.

Frankl’s behavior ratings were assigned independently and simultaneously by two calibrated pediatric dentists. Calibration was conducted using standardized case videos and consensus discussion, resulting in an inter-examiner reliability coefficient Kappa of 0.95. Any discrepancies were resolved through discussion at the time of the assessment.

In patients under premedication with oral Midazolam, a dosage of 0.3 mg/kg was administered 15 min before the procedure. The selected dose of 0.3 mg/kg oral Midazolam is consistent with established pediatric sedation guidelines and previous studies assessing its anxiolytic effects in dentistry [23,24]. This dosage has been widely documented as effective in reducing pre-procedural anxiety while maintaining patient safety, with minimal risk of adverse effects [24,25].

The groups were further stratified based on the type of dental procedure (invasive vs. non-invasive) to assess the influence of both sedation and procedural stress on salivary cortisol levels. Invasive treatments included treatments as restorations, extractions, pulpotomies, or crowns, and non-invasive treatments included initial visits, follow-ups, digital scans, sealants, and fluoride varnish.

**Figure 1:**
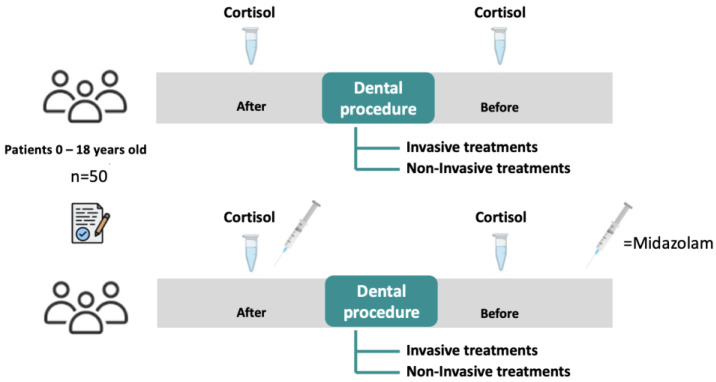
Patient groups for salivary cortisol assessment.

Saliva samples were collected before each treatment and immediately after the completion of the dental procedure, with parental consent (Figure 1).

This was a non-randomized, parallel-group, pre–post quasi-experimental pilot study. Due to the clinical allocation of premedication, all patients with definitely negative Frankl behavior received oral Midazolam, whereas patients with positive Frankl behavior were treated without sedation. This resulted in perfect collinearity between behavior and medication, making it impossible to model the independent effects of each factor. Consequently, statistical analyses should be interpreted as pragmatic comparisons of clinical management strategies (Midazolam in definitely negative children vs. no sedation in positive children) rather than as estimates of the isolated drug effect. The study followed the Strengthening the Reporting of Observational Studies in Epidemiology (STROBE) checklist.

### 2.2. Inclusion Criteria

Pediatric patients attending the clinic at the Catholic University of Valencia, aged under 18 years, diagnosed with Autism Spectrum Disorder (ASD), requiring non-invasive treatments (e.g., initial visits, follow-ups, digital scans, sealants, fluoride varnish), requiring invasive treatments (e.g., restorations, extractions, pulpotomies, crowns), exhibiting positive or definitely positive behavior according to the Frankl scale, or exhibiting definitely negative behavior according to the Frankl scale and requiring premedication with Midazolam.

### 2.3. Exclusion Criteria

Patients with known allergies to premedication drugs (Midazolam), medical conditions preventing active participation, or those previously included in this study. Children with ASD were included due to their higher prevalence of dental anxiety and behavioral management challenges, which make them a relevant population for evaluating anxiolytic interventions.

### 2.4. Variable Definitions

The Independent Variable (IV) was the Frankl behavior rating [26,27]. The Dependent Variable (DV) was salivary cortisol levels. Controlled Variables included premedication with Midazolam, age, gender, and type of dental treatment (invasive or non-invasive treatments). Confounding Variables encompassed general anxiety level, prior dental experiences, previous interactions with dental staff, overall health, and clinical environment.

### 2.5. Ethical Considerations

This study was conducted in accordance with the ethical guidelines of the Declaration of Helsinki (1964) and with approval from the Ethical Committee of Catholic University of Valencia, protocol code UCV/2022-2023/095.

### 2.6. Research Procedure

Informed consent was obtained from parents or legal guardians prior to saliva sample collection, in accordance with ethical guidelines. Saliva samples were collected only after obtaining consent, and parents were informed of their right to withdraw at any time. Saliva samples were collected upon arrival at the clinic (prior to the administration of Midazolam in premedicated cases) and after the clinical procedures were completed.

To reduce the effect of circadian rhythm on cortisol levels, all dental appointments were scheduled in the morning hours. However, no independent, procedure-free baseline measurement of salivary cortisol was obtained prior to the study visit, so individual baseline variability could not be controlled. Samples contaminated with blood or food debris were excluded from analysis; a total of 2 samples were excluded (Midazolam group: 1, control group: 2), with similar exclusion rates between groups.

### 2.7. Saliva Sample Collection

The working area was cleaned and disinfected before sample collection. Researchers used disposable gloves to minimize contamination risk. Children were given a disposable plastic cup for saliva collection. The procedure was clearly explained to participants to minimize the risk of sample contamination by food, blood, or other external substances. Saliva was collected in a calm environment, transferred to Eppendorf tubes using pipettes, and stored at −20 °C to maintain sample integrity. Post-treatment samples were visually inspected and excluded if contaminated with blood, particularly in invasive procedures, where this risk was higher.

### 2.8. Laboratory Analysis

Salivary cortisol was measured using a competitive enzyme-linked immunosorbent assay (ELISA) kit (Salimetrics^®^, State College, PA, USA) following the manufacturer’s protocol. Reagent preparation involved equilibrating reagents to room temperature (18–25 °C), preparing the wash solution (1×) by diluting 10× with deionized water, and preparing cortisol-HRP by mixing 10 µL of cortisol-HRP concentrate with 1 mL incubation buffer. The assay procedure included adding saliva samples to a 96-well plate pre-coated with anti-IgG cortisol, incubating with cortisol-HRP at 37 °C, adding TMB substrate solution and incubating at 37 °C, stopping the reaction with stop solution, and measuring absorbance at 450 nm. Calculation involved subtracting the average background absorbance from each calibration curve point and sample, and interpolating sample values on the standard curve to obtain cortisol concentrations in ng/mL. The procedure involved careful preparation, incubation, and optical density measurements using a Multiskan Spectrum spectrophotometer (Thermo Fisher Scientific Inc., Waltham, MA, USA) to quantify salivary cortisol concentrations.

### 2.9. Statistical Analysis

Inferential comparisons of salivary cortisol levels were conducted among different patient groups. Mean cortisol levels were compared before and after dental treatment between premedicated (Midazolam) and non-premedicated patients, as well as between those undergoing invasive and non-invasive treatments. Student’s *t*-tests for independent samples were employed to assess differences between groups, while non-parametric tests were used when normality assumptions were not met. Statistical significance was determined using *p*-values, confirming significant variations in cortisol levels post-treatment. No statistical differentiation was made between male and female patients due to the limited sample size, which precluded a reliable comparison between genders.

Data normality was assessed using the Shapiro–Wilk test. Parametric analyses (Student’s *t*-tests for independent or paired samples) were applied when data met normality assumptions; otherwise, non-parametric alternatives were used. Due to the small sample size and perfect collinearity between the Frankl behavior classification and Midazolam administration, multivariable analysis (e.g., ANCOVA) was not feasible. Therefore, results should be interpreted as unadjusted, reflecting pragmatic group comparisons.

## 3. Results

**Figure 2:**
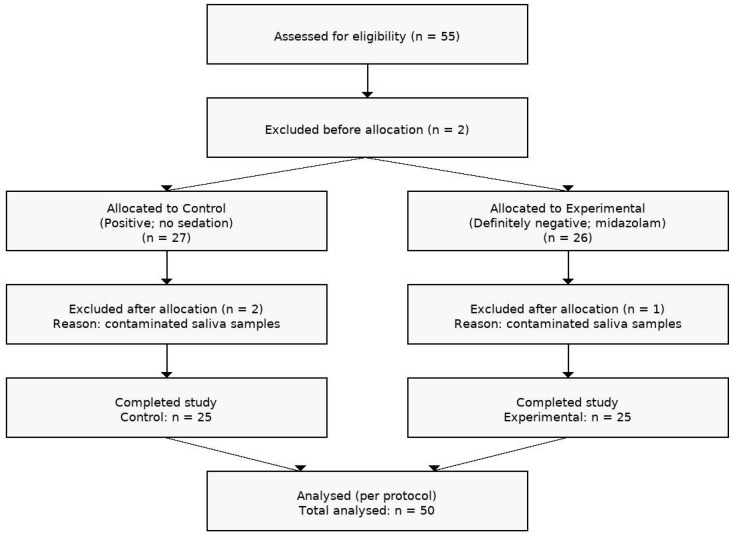
Participant flowchart showing the number of patients screened, excluded, assigned to each group, completing the study, and analyzed in the pilot study.

Fifty-five patients were assessed for eligibility; two were excluded before allocation and three after allocation. A total of 50 patients completed the study and were analyzed (Figure 2).

A total of 50 pediatric patients participated in the study, comprising 28 males and 22 females, with a mean age of 8 years and 2 months. Behavioral assessment was conducted using the Frankl scale. The study investigated salivary cortisol concentrations as a biological marker of stress before and after dental treatment. Comparisons were made between patients who received Midazolam premedication and those who did not, as well as between invasive and non-invasive procedures. Due to the limited sample size, potential differences between male and female participants were not analyzed independently.

### 3.1. General Comparison of Cortisol Levels

**Figure 3:**
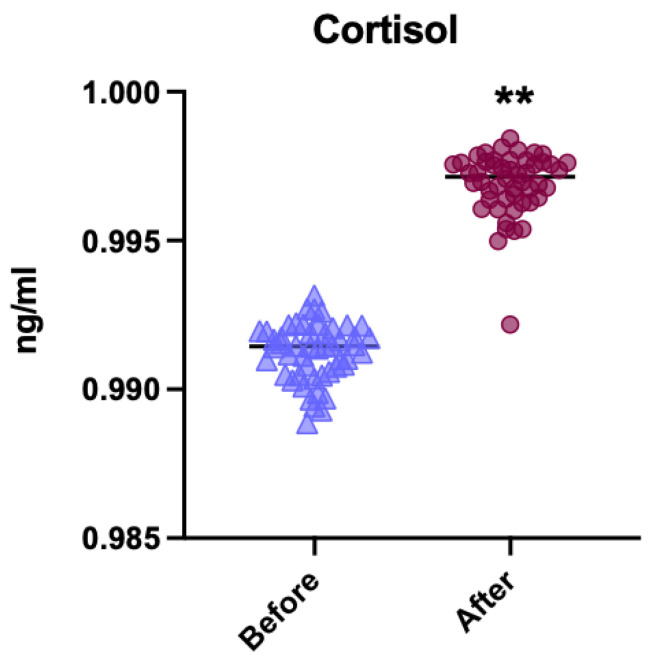
Comparison of cortisol levels before and after dental treatment (*n* = 25/group). ** *p* < 0.001.

The first comparison was performed on all patients, without distinguishing between different subgroups of treatment or premedication. After dental treatment, a significant increase in cortisol levels was observed, indicating higher stress levels associated with the dental procedure (Figure 3). This difference was statistically significant (*p* < 0.001).

### 3.2. Analysis of Cortisol Levels in Premedicated vs. Non-Premedicated Patients Before the Intervention

**Figure 4:**
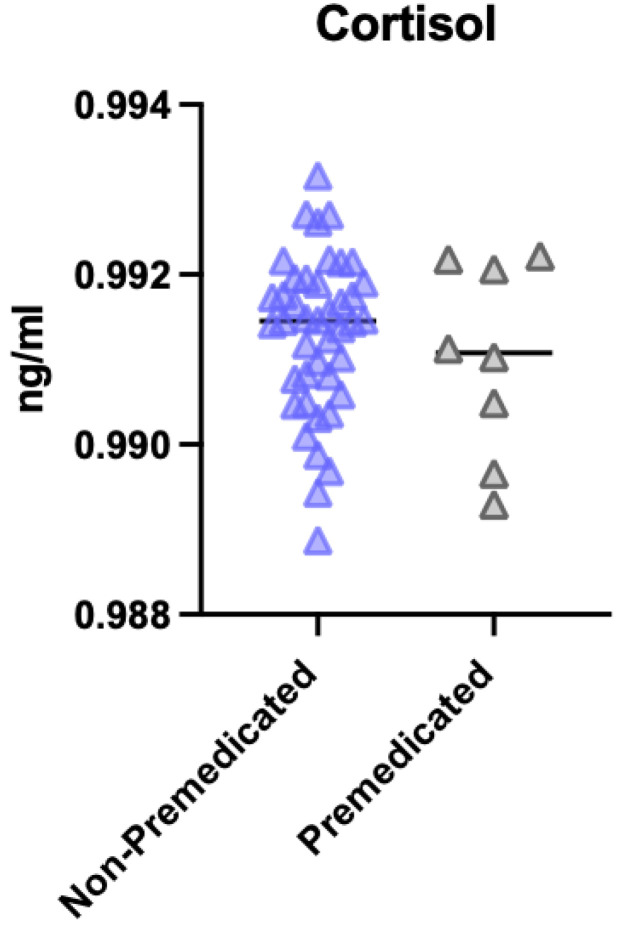
Cortisol levels before the intervention.

A subgroup analysis compared cortisol concentrations in patients who were administered Midazolam and those who were not before the intervention. Before treatment, no significant differences were observed in cortisol levels between the Midazolam and Control groups (d = 0.13). In contrast, after treatment, children premedicated with Midazolam showed significantly lower cortisol levels than controls, with a very large effect size (d = −1.52) (Figure 4).

### 3.3. Analysis of Cortisol Levels in Premedicated vs. Non-Premedicated Patients After the Intervention

**Figure 5:**
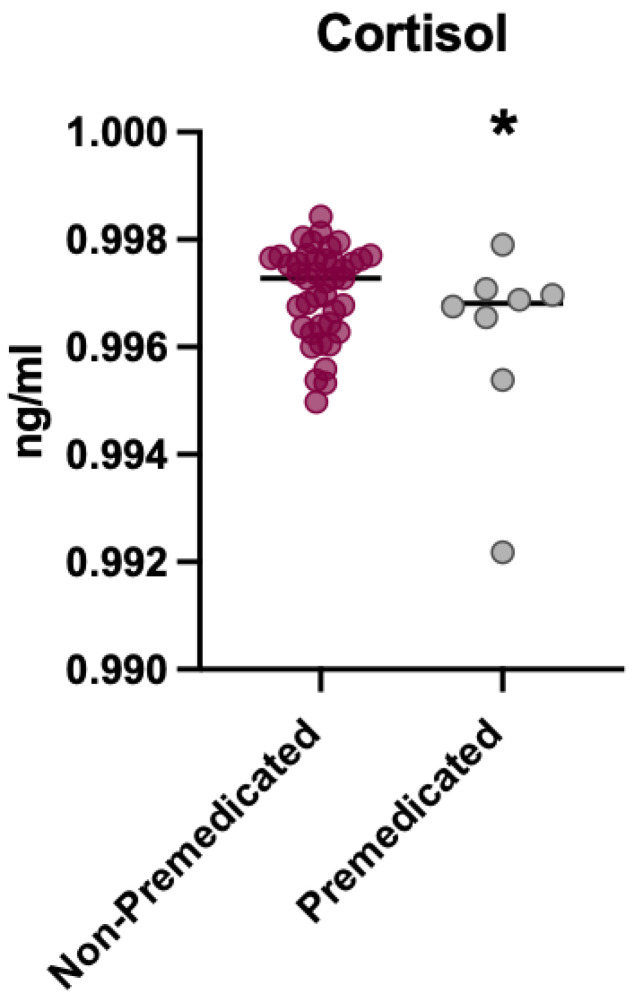
Cortisol levels after the intervention. * *p* < 0.05.

In contrast, after dental treatment, significant differences in cortisol levels were observed between patients who received Midazolam and those who did not (Figure 5). Cortisol concentrations were significantly lower in children premedicated with Midazolam compared to those who were not premedicated (*p* < 0.005).

### 3.4. Comparison of Cortisol Levels Between Invasive and Non-Invasive Treatments

**Figure 6:**
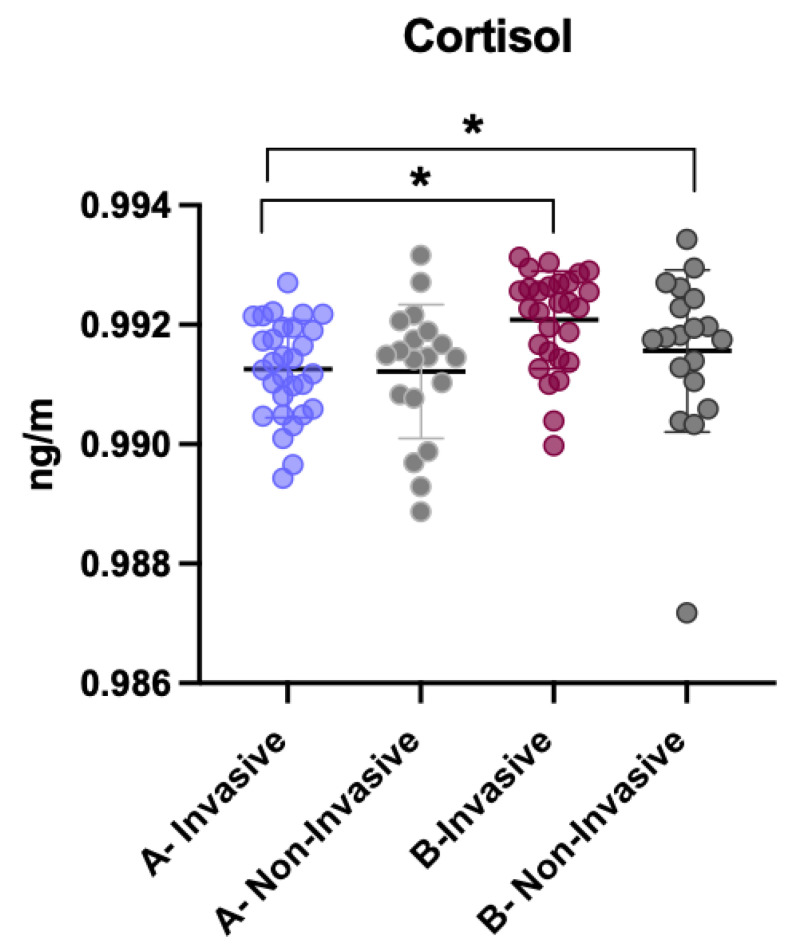
Comparison of cortisol levels between invasive and non-invasive treatments. * *p* < 0.05.

Additionally, cortisol levels were compared in patients undergoing invasive and non-invasive treatment, both before and after the intervention. Before treatment, there were no significant differences in cortisol levels between the two subgroups. However, after the intervention, patients who underwent invasive treatment had significantly higher cortisol levels compared to their levels before treatment (*p* < 0.001). In contrast, for non-invasive treatment, there was no statistically significant difference in cortisol levels before and after the intervention (Figure 6).

## 4. Discussion

The findings of this study, demonstrated through the quantification of salivary cortisol levels, strongly support the efficacy of Midazolam as an anxiolytic for children and adolescents with disruptive behavior according to the Frankl scale. Midazolam significantly reduced salivary cortisol levels, indicating its anxiety-reducing effect in these patients. Previous studies have extensively documented the efficacy of Midazolam in sedating children during dental procedures. For instance, Attri et al. concluded that oral Midazolam is a safe and effective sedation method in pediatric dentistry, significantly improving patient behavior and reducing perceived stress [28,29]. Similarly, Gomes et al. emphasized that Midazolam not only facilitates behavior control but also has a favorable safety profile [13,30].

It is important to note that, in this study, Midazolam administration was fully aligned with the Frankl behavior classification: all definitely negative patients received the drug, and all positive patients did not. This design choice, made for ethical and clinical reasons, creates perfect overlap between behavior and medication, which prevents disentangling the independent contribution of each. Therefore, the observed differences in cortisol levels should be understood as the outcome of two combined factors—behavioral profile and sedation strategy—rather than as the effect of Midazolam alone.

The choice to include children with positive Frankl behavior as the comparison group was based on the pragmatic clinical reality that such patients do not typically require sedation. However, this design choice inherently limits the ability to isolate the anxiolytic effect of Midazolam. By definition, children with positive behavior tend to be more cooperative and less anxious, which is generally associated with lower baseline cortisol levels. Consequently, differences in post-treatment cortisol between groups may partly reflect these baseline behavioral differences rather than the pharmacological effect of Midazolam alone.

The inclusion of both Frankl definitely negative and positive children in our study aimed to provide a comparative analysis of stress responses in different behavioral profiles. While focusing exclusively on definitely negative children could have provided a more targeted cortisol assessment, incorporating a control group allowed us to evaluate the extent to which Midazolam modulates stress in highly anxious patients versus those with naturally cooperative behavior. This broader comparison strengthens the clinical relevance of our findings and highlights the differential effects of sedation across various pediatric behavioral responses.

While salivary cortisol is a widely accepted biomarker for physiological stress, its levels can be influenced by circadian rhythm, acute environmental factors, and individual baseline variability. In our study, we minimized circadian effects by collecting all samples in the morning, but we did not obtain a procedure-free baseline for each participant. This limitation may have introduced variability unrelated to the dental intervention. Additionally, a small number of samples were excluded due to contamination, though exclusion rates were similar in both groups. Future studies could strengthen validity by including additional biomarkers (e.g., salivary alpha-amylase) and objective behavioral assessments to complement cortisol measurements.

The measurement of salivary cortisol levels is considered a reliable and non-invasive method for assessing stress levels in various clinical situations. This study confirmed that salivary cortisol concentrations significantly decreased after the administration of Midazolam, which aligns with the findings of other studies that used salivary cortisol as a stress index in dental practice [5]. In 2023, Moterane et al. obtained similar results, showing that premedication with Midazolam reduced salivary cortisol levels, thus confirming the utility of this biomarker for evaluating stress levels [15]. These results are consistent with the study by Gomes et al., which demonstrated that oral Midazolam could reduce cortisol levels under local anesthesia in children, thereby supporting its role in stress regulation in the clinical setting [13,29].

The literature has also documented a significant increase in salivary cortisol levels during more stressful dental procedures and intravenous administrations [25]. Similarly, the findings obtained in the present study showed a significant decrease in salivary cortisol concentration after the administration of Midazolam [2,30].

A systematic review conducted in 2014 highlighted the use of salivary cortisol as an indicator of physiological stress in both children and adults, demonstrating the utility of this biomarker in dental practice [17]. Comparing the results obtained in our study, the current literature consistently supports the use of conscious sedation with Midazolam as an effective strategy for reducing stress in pediatric patients, especially those classified as definitely negative according to the Frankl scale or exhibiting disruptive and uncooperative behavior during dental procedures.

A major limitation of this study is the confounding between the Frankl behavior rating and Midazolam premedication. Because all definitely negative patients received Midazolam and all positive patients did not, it was not possible to statistically separate the independent effects of behavior and medication. The findings, therefore, represent associative differences between two clinical management strategies rather than causal effects of the drug itself. Future research should include a subgroup of definitely negative patients without sedation, when ethically feasible, to isolate these effects. One potential strategy to ethically include a non-sedated definitely negative group would be the use of observational baselines during familiarization or acclimatization visits, in which no invasive procedures are performed. Collecting salivary cortisol samples in this context could provide a non-interventional reference point while avoiding unnecessary distress. In addition, gradual desensitization or non-pharmacological behavioral techniques could be applied in parallel, ensuring that children are not exposed to undue discomfort. Such approaches may allow future studies to better disentangle the effects of behavioral profile from pharmacological intervention while maintaining high ethical standards.

While the study provides valuable evidence supporting the efficacy of Midazolam and the use of salivary cortisol as a stress biomarker, several limitations must be acknowledged. Although salivary cortisol is widely recognized as a reliable indicator of physiological stress, its levels can be influenced by various physiological and environmental factors. This highlights the potential benefit of incorporating additional biomarkers to achieve a more comprehensive assessment of stress. Furthermore, the study investigated only one type of sedative (oral Midazolam), which may limit the generalizability of the findings to other pharmacological agents with differing mechanisms of action or efficacy profiles.

Another important limitation concerns the comparisons made between invasive and non-invasive procedures. These analyses did not control for confounding variables such as the Frankl behavior classification or the administration of Midazolam, which may have influenced cortisol responses independently of the treatment type. Future research should incorporate stratification or statistical adjustment for these factors to more accurately isolate the effects of procedural invasiveness.

The inclusion of a wide pediatric age range was intentional, aimed at capturing potential developmental differences in stress response, as younger children often present with heightened dental anxiety. However, due to the limited sample size characteristic of pilot studies, the statistical power was insufficient to conduct meaningful subgroup analyses based on age, sex, or other stratified variables. Larger-scale studies are needed to confirm these preliminary findings and to allow for more robust multivariable analyses. The study did not evaluate or adjust for potentially confounding variables such as baseline general anxiety, prior dental treatment experiences, or familiarity with dental staff, which may have influenced the cortisol responses observed.

## 5. Conclusions

The results of this pilot study suggest that oral Midazolam administration in pediatric patients with “definitely negative” behavior according to the Frankl scale could be associated with lower salivary cortisol levels after dental treatment. However, due to the limited sample size and quasi-experimental design, these findings should be considered preliminary. Larger studies with controlled designs are needed to confirm these results and more precisely explore the influence of medication, baseline behavior, and other confounding factors on stress response. The study highlights the importance of considering psychological factors and physiological responses in pediatric dental care, suggesting future research should explore broader sample sizes, incorporate multiple biomarkers, and evaluate different premedication types for further optimization.

## Figures and Tables

**Table 1:** Study demographics.

Characteristic	Midazolam Group (*n* = 25)	Control Group (*n* = 25)	Total (*n* = 50)
Age, mean ± SD (years)	8.1 ± 2.3	8.3 ± 2.1	8.2 ± 2.2
Sex, *n* (%)			
- Male	14 (56%)	14 (56%)	28 (56%)
- Female	11 (44%)	11 (44%)	22 (44%)
Type of procedure, *n* (%)			
- Invasive	15 (60%)	12 (48%)	27 (54%)
- Non-invasive	10 (40%)	13 (52%)	23 (46%)

## Data Availability

The data supporting the findings of this study are available from the corresponding author upon reasonable request. Due to institutional policy, the datasets are not publicly available.
